# Supplementary material for: Overexpression of OsFTL10 induces early flowering and improves drought tolerance in Oryza sativa L
Source: PeerJ. 2019 Feb 12;7:e6422. doi: 10.7717/peerj.6422 (PMC6376957; doi:10.7717/peerj.6422)
Supplement: Supplemental Information 8 — About 2 kb DNA sequences upstream of OsFTL10 were download and analyzed http://www.dna.affrc.go.jp/PLACE/signalscan.html. [file peerj-07-6422-s009.docx]

Analyses of cis elements in the promoter of OsFTL10

1. Sequence of the promoter of OsFTL10

>Chr5:25669369..25671369

ttgatctgtagggctagctagcgcactatgagtcggggtaactagtatccaagcataaac

tatttatatggtgaatgatgagggttgtcttgtacattcacctttcaaaagttagacatc

atgtgatgcgtcacccatacgaaactgtttctttgaattggtattcatatttgttgatta

gattatgggacctatggattagtcatgcatcagatttctttgacctctaaggttgagcta

ccctgcccatcagagattggtagcatagtttgtgtctactggagcaagctttagtggtta

gcacaaacagctgttcttttggtcatatcctctcattgcagacgacaaagatcatgcttc

ataacttaattgtatacttgtgtcgaacattggggtgtacttaatgtgttctgattctca

ctgcaacaccgtatctgagcagtagacatctaggaatattttttgccttgcctttcttgc

atctaatgcatttgtagaaagtgtctgtttatccttttgcgaacaccaaggaacttaaat

agcttatttacttaagttattttaggggttacacaatggtactttttttaataggcacaa

tggtactttaggagcaagacaaatgtatagcaagtttgttgcggctcatgtgcgatgagc

ttgtgctgtaacatttatttacaaggtggattgtttctattatttggacctgtgaaatat

catatgacaatttattttggaacatggacatcattcttttgttattgtcaaaaaagtgag

atcgctgtgtgcatgttcccaaggctcaaagttagtttatgtgttatgttgcttgatcgc

tgtcatattttggcaccatcttgtttctatttcatgtttctttttcttcagtatgcaaat

ggcattcttcaatatgtactctaacatcactggagtttctgctgcactgtttgttcccat

attgacttgattagcatgatttaagtttccacaaagtgtagcagtaaaatgcatcatttg

cttcatcatgcatgagtaaaaattggcttgattgcctgcggtttatcttgtacgctaccc

gcatagggtctcaatacaggttattaactactttacatatattttcatcgggcacaaagg

aaaaaaacaattcaaggttgtcatttggaagtcctgtaccactttcttcacttcaggata

aggattctatttctaccatttgctaggaaagcagattcttcttgtaccttcttcacatgg

agcaaaaaatgacccgaggattctagttacttcctctgtttcagactataagtcgttttg

actttagtcaaagttaaactgctccaaatttatagataaactgctccaagtttatagata

aaaagtcaaaacaaactataatctaaaacggagggagtaacgttttttgaaggcacatgc

ctgatttgattgtcagatgctcaatactataatgcaaggaaagctgtgctctatggtctt

cagtaactttgatatgccaaagaggaggacttttcgtcataaaaaaggtattacactaca

tacaaggaaaaactgaacaatcctttttaagcttttattgaatggacaatattcgtcctt

tgatgttttcttaaagggagaagggaggggggcatgttatctttctagatgttgctatca

cagctttgataataaaaatactatgacagcttcgataataaaaatactatgttatcaatc

atgtgtggtcattggccaaccagatgttggcctatgccatgtaactagaaatcgtctcac

taagatatgacctcttaacataatgctatatttactcaattgcatggaattatggtgtta

actgtttgtgtgatgtcactgttgcatgtatcaaccatacactctgtcatgtgtccagtt

atgttgcaactaaaccagatggcatataggggggctttatgtcttttgcatgagatttgt

gtatgaccgattgataagccc

2. Prediction of Cis elements in *OsFTL10* promoter

<http://www.dna.affrc.go.jp/PLACE/signalscan.html>

| Signaling pathway | Cis-elements | Code | Copies |
| --- | --- | --- | --- |
| Salt | GAAAAA | S000453 | 3 |
| Drought | ACGT | S000415 | 2 |
|  | WAACCA | S000408 | 2 |
|  | YAACKG | S000409 | 2 |
|  | CNGTTR | S000176 | 3 |
|  | CATGTG | S000413 | 6 |
|  | CACATG | S000174 | 6 |
| Low temperature | CCGAC | S000153 | 1 |
|  | CANNTG | S000407 | 3 |
| Light | TGACG | S000024 | 2 |
|  | GGGCC | S000483 | 1 |
|  | GATAAG | S000124 | 2 |
|  | ACTTTG | S000383 | 4 |
| Auxin | KGTCCCAT | S000026 | 1 |
|  | GATAA | S000199 | 14 |
|  | GGTCCCAT | S000360 | 1 |
|  | CATATG | S000370 | 2 |
|  | GATAAGR | S000424 | 1 |
|  | ACTTTA | S000273 | 3 |
| Abscisic Acid | CATGCATG | S000102 | 2 |
|  | ACACNNG | S000292 | 3 |
|  | CTAACCA | S000175 | 1 |
|  | CATGCA | S000264 | 7 |
| Salicylic acid | TTGAC | S000390 | 8 |
| Ethylene | AWTTCAAA | S000037 | 1 |
| GA | TAACAAR | S000439 | 1 |
|  | TAACAAA | S000181 | 11 |
|  | TTTTTTCC | S000298 | 1 |
|  | CCTTTT | S000259 | 3 |
| Endosperm | TGHAAARK | S000122 | 2 |
|  | CNAACAC | S000148 | 1 |
| Anther specific | AGAAA | S000245 | 14 |
|  | AGGTCA | S000254 | 2 |
| Sucrose | TTATCC | S000470 | 3 |
| Copper | GTAC | S000493 | 18 |
